# 10% Higher Rowing Power Outputs After Flexion-Extension-Cycle Compared to an Isolated Concentric Contraction in Sub-Elite Rowers

**DOI:** 10.3389/fphys.2020.00521

**Published:** 2020-06-17

**Authors:** Steffen Held, Tobias Siebert, Lars Donath

**Affiliations:** ^1^Department of Intervention Research in Exercise Training, German Sport University Cologne, Cologne, Germany; ^2^Department of Motion and Exercise Science, University of Stuttgart, Stuttgart, Germany

**Keywords:** SSC, ergometer, motion capture, concentric, eccentric, force enhancement, muscle, potentiation

## Abstract

The resulting muscular performance is considered notably higher during a stretch shortening cycle (SSC) compared to an isolated concentric contraction. Thus, the present study examined the occurrence and magnitude of rowing performance enhancement after a flexion–extension cycle (FEC) of the legs compared to both concentric contractions only and isometric pre-contraction. Therefore, 31 sub-elite male rowers (age: 25 ± 6 years, height: 1.90 ± 0.02 m, weight: 91 ± 10 kg, weekly training volume: 11.4 ± 5.3 h/week, rowing experience: 7.1 ± 2.7 years) randomly completed (a) isolated concentric rowing strokes (DRIVE), (b) single FEC-type rowing strokes (SLIDE-DRIVE), and (c) rowing strokes with an isometric pre-contraction (ISO-DRIVE). The resulting rowing power (P_row_), leg power (P_leg_), and work per stroke (WPS) were recorded using motion-capturing, force, and rotation sensors. Comparison of DRIVE and SLIDE-DRIVE revealed significantly (*p* < 0.05) higher P_row_ (+11.8 ± 14.0%), P_leg_ (+19.6 ± 26.7%), and WPS (+9.9 ± 10.5%) during SLIDE-DRIVE. Compared to ISO-DRIVE, P_leg_ (+9.8 ± 26.6%) and WPS (+6.1 ± 6.7%) are again significantly (*p* < 0.05) higher for SLIDE-DRIVE. In conclusion, notably higher work and power outputs (compared to an isolated concentric contraction) during FEC rowing referred to an underlying SSC. Future ultrasound studies should elucidate whether a real SSC on the muscle tendon unit level account for these performance enhancements.

## Introduction

The sequence of stretching and subsequent contraction of a muscle tenon unit (MTU) is considered a stretching shortening cycle (SSC) (Komi, 2003). The resulting muscular force, work, and power during an SSC enable up to 50% higher power output values compared to isolated concentric contractions (Cavagna et al., 1968; Bosco et al., 1987; Gregor et al., 1988). Increased muscular efficiency and decreased metabolic costs have been discussed to account for these findings (Dawson and Taylor, 1973; Aura and Komi, 1986). The increased muscle performance during SSC is, however, still not completely understood (Seiberl et al., 2015). The power enhancement during an SSC can be mainly attributed to (a) the storage and release of elastic energy (Kubo et al., 1999; Bojsen-Møller et al., 2005), (b) stretch-induced contractility enhancement (Rode et al., 2009; Seiberl et al., 2015), and (c) reflex activity and time-to-peak force (Schenau et al., 1997a, b).

**FIGURE 1:**
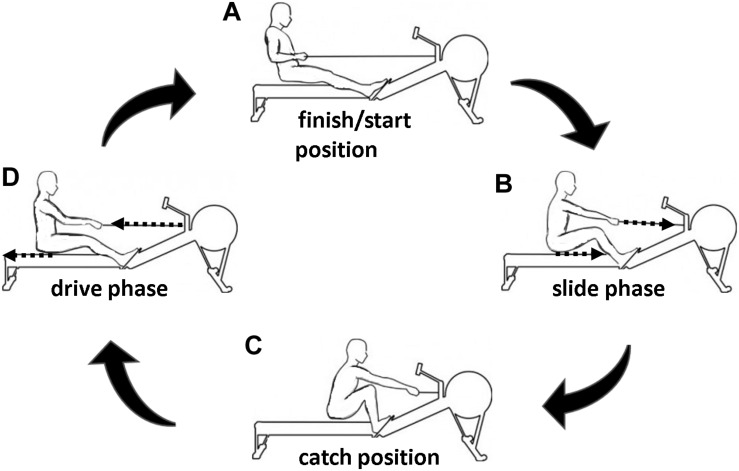
Exemplary representation of a rowing cycle separated into different phases: slide **(B)** and drive **(D)** phase. The finish/start **(A)** and catch **(C)** position are the turning points of the extension and flexion phase, respectively.

In this regard, the rowing cycle can be classified into a propulsive phase (drive, see Figure 1D) and a gliding phase (slide, see Figure 1B). During one rowing cycle, the legs are firstly undergoing a flexion (slide) followed by an extension pattern (drive). This flexion–extension cycle (FEC) movement can be performed in rowing as fast as in countermovement jumps (Held et al., 2019, 2020). The leg extensor muscle activity (rectus femoris, vastus medialis, and vastus lateralis) during the late slide phase prior to the onset of a new rowing stroke was detected (Janshen et al., 2009; Guével et al., 2011; Turpin et al., 2011; Fleming et al., 2014; Shaharudin et al., 2014; Held et al., 2020). Accordingly, the combination of flexion (slide) and extension (drive) of legs (potentially corresponding to a stretching and contraction of leg extensor muscles) in rowing can be defined as a FEC.

As more than 90% of the annual rowing training is completed at low stroke rates (Steinacker, 1993; Guellich et al., 2009; Bourgois et al., 2014), aspects of reactive forces are rarely considered in rowing training (Held et al., 2019, 2020). These low stroke rates (about 18 spm) are characterized by approximately four times the duration of the slide phase during a 2,000-m rowing competition (about 36 spm or higher) (Kleshnev, 2016; Held et al., 2019) and showed no leg extensor muscle (vastus medialis) activity during the late slide phase prior to the onset of a new rowing stroke (Held et al., 2020). This imbalance between training and competition requirements seems unsuitable due to the force–velocity relation of the muscle (van Soest and Casius, 2000) and the SSC (Komi, 2003).

Against this background, the present study was conceptualized and conducted in order to elucidate whether rowing enables force, work, and power enhancement (as described above) during FEC-type rowing compared to isolated concentric muscle actions (drive phase only, Figures 1C,D) comparable to those expected in SSC. The underlying design was based on the assumption that training a sport-specific muscle action is required and has been repeatedly emphasized (Gollhofer et al., 1987; Komi, 2003; Nicol et al., 2006). Therefore, we aimed at investigating the occurrence and magnitude of force, work, and power outputs during FEC-type rowing compared to isolated concentric rowing and concentric rowing with isometric precontraction on the rowing ergometer. We assume that the general force, work, and power enhancement of FEC-type rowing are crucial and meaningful. Finally, the resulting data would have an impact on the conceptualization of rowing-specific testing and training by paying more attention to reactive force abilities.

## Materials and Methods

### Participants

Thirty-one sub-elite male rowers (age: 25 ± 6 years, height: 1.90 ± 0.04 m, weight: 91 ± 10 kg, 2,000-m ergometer Time Trial mean power: 374 ± 74 W, weekly training volume: 11.4 ± 5.3 h/week, rowing experience: 7.1 ± 2.7 years) were enrolled in this randomized controlled crossover trial. The inclusion criteria were as follows: at least 5 years of rowing competition experience and at least rowers on the national level with no health complaints and impairments. After providing all relevant study information, informed consent was requested from all athletes prior to the start of the study. The study protocol complied with the Declaration of Helsinki and has been previously approved by the local ethical committee (001/2019), fulfilling the international ethical standards (Harriss and Atkinson, 2015).

### Study Design

After a standardized 15-min warm-up program (10-min rowing at a low intensity/heart rate, which corresponds to a blood lactate concentration <2 mmol/L and about three practice trials), the participants performed five isolated concentric rowing strokes (DRIVE, see Figures 1C to 1A), five single rowing strokes with isometric precontraction (ISO-DRIVE, see Figures 1C to 1A), and five single FEC-type rowing strokes (SLIDE-DRIVE, see Figure 1) in a randomized order. Since the DRIVE measurement was started with non-activated muscle, the muscle was already pre-activated in the SLIDE-DRIVE-measurement at the beginning of the concentric phase (Janshen et al., 2009; Guével et al., 2011; Turpin et al., 2011; Fleming et al., 2014; Shaharudin et al., 2014; Held et al., 2020). Accordingly, measurements with an isometric precontraction (ISO-DRIVE) were additionally performed in order to observe the different starting conditions of the DRIVE and SLIDE-DRIVE trials. The DRIVE measurements started at the catch position (see Figure 1C) and consisted only of the drive phase (see Figure 1D) until the finish position (see Figure 1A). During the ISO-DRIVE measurements, an additional 3-s-lasting isometric precontraction was performed with maximal efforts. Thereby, the rowing handle was fixed at the catch position (see Figure 1C) using a hook, which was released upon the start signal. The SLIDE-DRIVE measurements comprise a full rowing cycle (slide and drive phase; see Figure 1), starting at the finish position. The participants received the instructions to generate maximum power for each measurement trial. The mean values of the three rowing strokes with the highest power outputs (of the five attempts) for each rowing condition were included into further analyses. Between all rowing strokes, a break of 2 min was guaranteed. The flywheel of the rowing ergometer was still standing at the start of the drive phase during all rowing conditions (DRIVE, ISO-DRIVE, and SLIDE-DRIVE). A complete familiarization session (consisting of 10 DRIVE, ISO-DRIVE, and SLIDE-DRIVE rowing strokes) was completed 1 week before the measurement, and the athletes were asked to refrain from any strenuous activity 24 h prior to each assessment condition.

### Data Collection

All tests were performed on a wind-braked rowing ergometer (Concept2/Type D, Morrisville, NC, United States). The ergometer was additionally equipped with the FES Ruderergo-System [Institut für Forschung und Entwicklung von Sportgeräten (FES), Berlin, Germany] using a load cell for handle force (F_drive_) measurement (Type KM26z; ME-Meßsysteme GmbH, Hennigsdorf, Germany) placed between the chain and the handlebar. Therewith, precise measurements of mechanical power were enabled. Since the load cell was placed between the chain and the handlebar, the forces of each isometric precontraction cannot be detected. As there is no handlebar movement during this isometric precontraction, no mechanical power output was generated during this phase. Accordingly, the used setup is considered suitable for the investigation of power outputs during all dynamic rowing conditions (Treff et al., 2018). An incremental encoder (ERN 1020/250 01-03; Heidenhain, Traunreut, Germany) was placed on the rotation axis of the flywheel to measure the displacement of the handlebar. The error of measurement of the FES setup was equal to or smaller than 1.5% (Treff et al., 2018). In addition to the kinematic assessment, a motion capturing system was employed (Rienks et al., 2020): The entire measurement was video-captured using 120 fps. The employed camera (Type Hero 5, GoPro, San Mateo, CA, United States) was placed in the middle of the rowing ergometer (90°-angled distance 3 m, height 0.5 m). The seat and handle positions (marked with luminous markers) were captured using a motion-capture software (Tracker, open-source physics, Boston, MA, United States). For the calibration of the motion-capturing system, a coordinate system including proper scaling was defined: In this context, a known length (1-m scale, marked on the seat track of the rowing ergometer) was marked in the video with the help of the motion-capturing software. This calibration scale was located at the same level (distance to the camera) as the handle and seat movement. In addition, the reference coordinate was uniformly placed in the axis of the rotation of the flywheel. The accuracy of the method was five pixels, which corresponds to less than 0.01 m at the current setup (Suleder, 2010). This accuracy was confirmed by comparing the handle displacement data of the FES setup (error of measurement <2%) with the motion-capturing data. Based on this motion-capturing, the length of the seat motion (L_slide_) was recorded. Then, the speed of the seat (corresponding to leg extension or shortening velocity, vleg) was subsequently determined as the derivation of (time-dependent) seat position. Mechanical work per stroke (WPS) and mechanical rowing power (P_row_) were calculated based on the data of the FES Ruderergo System. In addition, the maximum force during the drive (F_max_), the force at the catch position (F_0_), the drive time (T_drive_), the length of the drive (L_drive_), and the handle speed during the drive (v_drive_) were determined. Rowing power (P_row_) was calculated by multiplying the handle force F_drive_(t) by velocity v_drive_ (t) (Fukunaga et al., 1986; Dal-Monte and Komo, 1989; Zatsiorsky and Yakunin, 1991). The proportion of leg power (P_leg_) on the total P_row_ was further determined based on multiplying F_drive_(t) by leg (seat) movement speed v_leg_(t) (Kleshnev, 2016; Held et al., 2019). Based on the current measurements, DRIVE (*r* = 0.998, *p* < 0.001), ISO-DRIVE (*r* = 0.970, *p* < 0.001), and SLIDE (*r* = 0.994, *p* < 0.001) showed exclusively high split-half-reliability values.

### Statistics

Statistical analyses were performed using a statistic software package (IBM SPSS Statistics, Version 25.0, Armonk, NY, United States). All data are presented as group mean with standard deviation. All data were checked for normal distribution and variance homogeneity using the Kolmogorov–Smirnov and Levene tests, respectively. Separate repeated measurement analysis of variance (rANOVA) was applied for the different rowing conditions (DRIVE, ISO-DRIVE, and SLIDE-DRIVE) using P_row_, WPS, F_max_, F_0_, F_drive_(t), T_drive_, L_drive_, v_drive_, L_slide_, and P_leg_ as within-subject variables. In case of significant interaction effects, Bonferroni *post hoc* tests were subsequently computed for pairwise comparisons. To estimate the overall time and interaction effect sizes, η_p_^2^ were calculated with η_p_^2^ ≥ 0.01 indicating small, ≥0.059 medium, and ≥0.138 large effects (Cohen, 1988). Standardized mean group differences as a measure of pairwise effect size estimation were also calculated (SMD, trivial: *d* < 0.2, small: 0.2 ≤ *d* < 0.5, moderate: 0.5 ≤ *d* < 0.8, large *d* ≥ 0.8) (Cohen, 1988). Moreover, a *p*-value below 0.05 was considered statistically significant.

## Results

### Handle Forces as a Function of Handle Length and Speed

**FIGURE 2:**
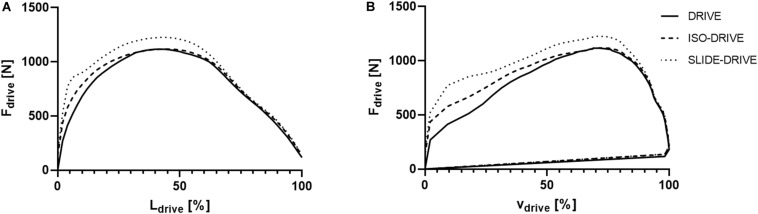
Representation of the average handle force (F_drive_) as a function of handle pathway length (length of drive: L_drive_; **A**) and as a function of handle speed during drive (v_drive_; **B**) for the concentric (DRIVE), isometric precontraction (ISO-DRIVE), and FEC type (SLIDE-DRIVE) rowing trials. The force length and force–speed graph are normalized to the length of the drive.

Figure 2A shows the averaged force–distance graphs for the DRIVE, ISO-DRIVE, and SLIDE-DRIVE conditions. In the catch position (L_drive_ = 0%) the SLIDE-DRIVE and ISO-DRIVE forces (F_0_) were obviously higher than the DRIVE force (*p* < 0.001, η_p_^2^ > 0.138) due to muscle preactivation. It is clearly visible that SLIDE-DRIVE forces F_drive_(t) are higher (*p* < 0.001, η_p_^2^ = 0.398) than DRIVE forces during almost the total rowing stroke (T_drive_). The averaged force–speed graphs of the DRIVE, ISO-DRIVE, and SLIDE-DRIVE measurements for the entire sample are displayed in Figure 2B. From visual inspection, the loop area (P_row_) increases from DRIVE to ISO-DRIVE to SLIDE-DRIVE, which is also confirmed by the following rANOVA results.

### Power, Work, and Force

**FIGURE 3:**
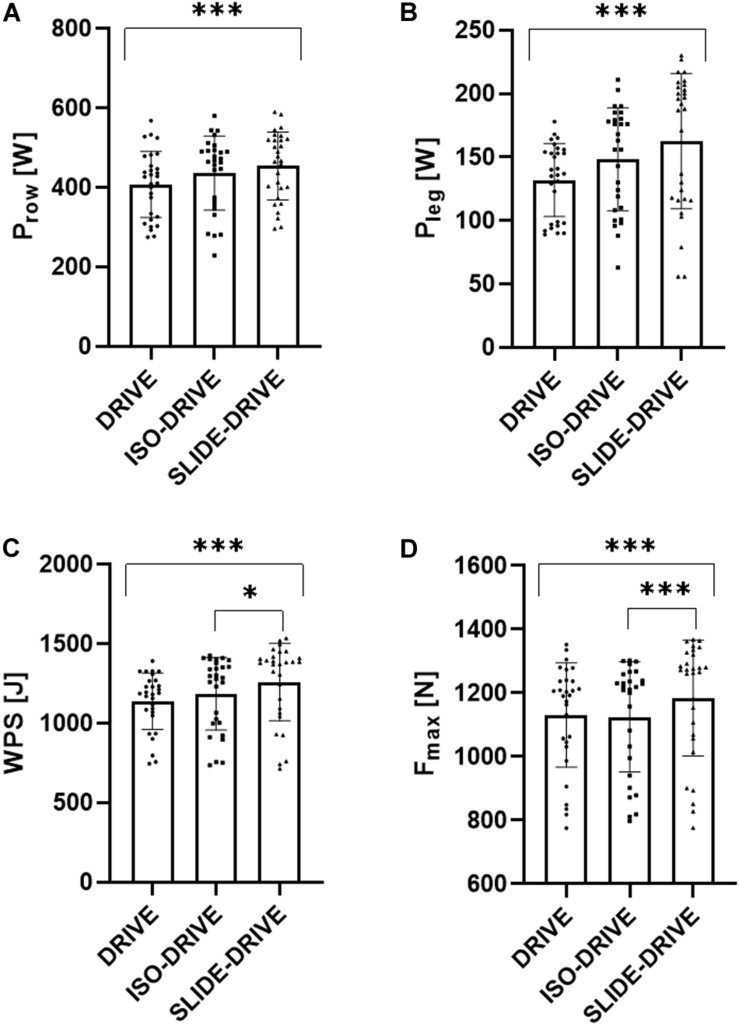
Representation of rowing power (P_row_, **A**), leg power (P_leg_, **B**), work per stroke (WPS, **C**), and maximum force (F_max_, **D**) results of the concentric (DRIVE), isometric precontraction (ISO-DRIVE), and FEC type (SLIDE-DRIVE) rowing trials (mean ± standard deviation). In addition, the significances of the post-hoc tests were presented (***p < 0.001; *p < 0.05).

The rANOVA yielded significant interaction effects (0.01 < *p* < 0.001; 0.214 < η_p_^2^ < 0.331) for the rowing conditions (DRIVE, ISO-DRIVE, SLIDE-DRIVE) regarding all parameters (P_row_, P_leg_, WPS, F_max_, L_drive_, L_slide_, and v_drive_), except for T_drive_ (*p* = 0.351; η_p_^2^ = 0.072). Subsequent pairwise *post hoc* testing showed a significant (*p* < 0.05) increase of P_row_ (see Figure 3A; +11.8 ± 14.0%, SMD = 0.290), P_leg_ (see Figure 3B; +19.6 ± 26.7%, SMD = 0.429), WPS (see Figure 3C; +9.9 ± 10.5%, SMD = 0.534), F_max_ (see Figure 3D; +4.4 ± 7.0%, SMD = 0.260), L_drive_ (+6.3 ± 4.8%, SMD = 0.552), and v_drive_ (+7.6 ± 6.0%, SMD = 0.889) between DRIVE and SLIDE-DRIVE measurements. Between ISO-DRIVE and SLIDE-DRIVE measurements, the *post hoc* tests show only significant (*p* < 0.05) increases in WPS (see Figure 3C; +6.1 ± 6.7%, SMD = 0.307), F_max_ (see Figure 3D; +5.0 ± 4.8%, SMD = 0.287), L_drive_ (+3.2 ± 5.2%, SMD = 0.210), and v_drive_ (+4.2 ± 4.0%, SMD = 0.587). In contrast, the *post hoc* tests show no significant (*p* > 0.05, SMD < 0.448) differences between DRIVE- and ISO-DRIVE measurements for all variables (P_row_, P_leg_, WPS, F_max_, L_drive_, L_slide_, and v_drive_). The descriptive data, the resulting effects of ANOVA, and the percentage increases for the DRIVE, ISO-DRIVE, and SLIDE-DRIVE measurements are presented in Supplementary Table S1.

## Discussion

The present randomized controlled crossover trial aimed at investigating whether maximum handle forces, WPS, P_row_, and P_leg_ differ depending on the applied rowing movement pattern. We intended to elucidate whether a flexion–extension cycle (FEC) leads to notably higher power outputs compared to a pure concentric movement. Therefore, single (purely) concentric rowing strokes (DRIVE, see Figures 1C to 1A), single FEC-type rowing strokes (SLIDE-DRIVE, see Figure 1), and rowing strokes with isometric precontraction (ISO-DRIVE, see Figures 1C to 1A) have been examined. Compared to purely concentric rowing (DRIVE), remarkably higher WPS, P_row_, and P_leg_ have been observed during ISO-DRIVE and SLIDE-DRIVE measurements (see Figure 3). Compared to ISO-DRIVE, these increases (from DRIVE to ISO-DRIVE) in P_row_, P_leg_, and WPS remain statistically insignificant (see Figure 3). A tendency toward higher values during ISO-DRIVE can be, however, partly explained by the following assumptions: In general, muscle activity and performance are higher (in particular) at the beginning of a concentric movement when preceded by an isometric precontraction (compared to purely concentric contractions) (Svantesson et al., 1994). Despite the fact that we did not measure muscle activity, we assume higher muscle activations at the beginning of a rowing stroke during the ISO-DRIVE condition, compared to almost non-activated muscles in the DRIVE condition, which results in higher handle forces at the start of the rowing stroke. The SLIDE-DRIVE measurement revealed a significantly and notably higher maximum handle force (compared to DRIVE). Consequently, muscular P_row_, P_leg_, and WPS during FEC-type rowing (SLIDE-DRIVE) elicited between 10 and 20% higher values compared to isolated concentric (DRIVE) rowing strokes. These results are in line with earlier findings pointing to performance (force, work, and power) enhancements within an SSC in isolated muscle preparations with constant electrical stimulation (Cavagna et al., 1968), in animal experiments with natural and variable muscle activation (Gregor et al., 1988), and during maximal voluntary SSC actions of human muscles (Cavagna et al., 1968; Aura and Komi, 1986; Bosco et al., 1987). Overall, the present study revealed that rowing showed similar performance enhancements like other reactive (SSC) sports movements: A vertical jump preceded by a countermovement (SSC) will increase vertical displacement above a squat jump (concentric only) (Bosco et al., 1987; Bobbert et al., 1996; Bobbert and Casius, 2005). Similarly, a windup movement in throwing (SSC) resulted in an increased power output (Newton et al., 1997). Consequently, the rowing cycle behaves like other SSC (sports) movement. Although we cannot clearly presume a real SSC without ultrasound verification, this increase in P_row_ and forces due to a potential SSC has been frequently linked to the storage and release of elastic energy (Kubo et al., 1999; Bojsen-Møller et al., 2005), stretch-induced contractility enhancement (Rode et al., 2009; Seiberl et al., 2015), reflex activity, and time-to-peak force (Schenau et al., 1997a, b). Since muscle reflexes and preactivation in rowing have been ruled out in a previous (surface) electromyographic (sEMG) study (Held et al., 2020), the storage (and delivery) of elastic energy (induced by muscle activation during the eccentric phase) and the stretch-induced increase in contractility during the concentric phase are most likely relevant contributors to the observed performance enhancement in rowing. In the context of rowing, it should be noted that in addition to P_row_, P_leg_, and WPS, also the total stroke length (L_drive_) increased from DRIVE to SLIDE-DRIVE. This could be an indication of a changed rowing strategy. Since the amount of leg movement (L_slide_) does not change between all conditions (DRIVE, ISO-DRIVE, SLIDE-DRIVE), the FEC seems to be unaffected. Nevertheless, the aspect of different stroke lengths for DRIVE, ISO-DRIVE, and SLIDE-DRIVE should be considered in further research.

Investigations revealed that force, work, and power increase during an SSC of up to 50% compared to isolated concentric contractions (Cavagna et al., 1968; Gregor et al., 1988); the observed performance (work and power) increase (about 10–20%) of flexion–extension contractions rowing is comparatively low. In contrast, jump-specific SSC showed a performance increase in the countermovement jumps compared to squat jumps of 18–30% (Bosco et al., 1987; Bobbert et al., 1996; Bobbert and Casius, 2005), which is closer to the observed performance enhancement in rowing. In this context, the maximum kinetic energy during a rowing slide is 60 ± 20% of the (maximum) potential energy for a (drop) jump (Held et al., 2020). As a consequence, the potential energy to be stored during rowing is notably lesser than in (drop) jumps. These differences may be key reasons for a smaller power enhancement during SLIDE-DRIVE rowing compared to jumping. Moreover, five subjects (equivalent to 13.5% of the entire sample) showed lower work and power outputs during the SLIDE-DRIVE measurement (compared to DRIVE and ISO-DRIVE measurements). These few poor responders might exhibit a deficiency of reactive force capabilities (motions in SSC). In general, numerous studies showed (Stojanović et al., 2017; Berton et al., 2018) that SSC performance can be increased mainly by reactive force capabilities, induced by adequate training (e.g., plyometrics). In the context of plyometric training in rowing, contradictory research results, however, exist: While one intervention study (*n* = 18, 4 weeks) revealed rowingspecific performance improvements through plyometric training (Egan-Shuttler et al., 2017), another intervention study (*n* = 24, 9 weeks) observed no rowing-specific performance improvements (Kramer et al., 1993). These contradictory findings might partly be explained by methodological issues. It has been recently shown that examinations of sEMG-activity of selected leg muscles (*m. vastus medialis* and *m. gastrocnemius medialis*) during single scull rowing showed no preactivation and no reflex activity, which implicate that any forms of muscle action in the fast SSC domain (e.g., induced during drop jump) do not reflect discipline-specific muscle actions and could hamper rowing performance enhancement during training and competitions (Held et al., 2020). Moreover, both studies did not differentiate participants due to their reactive force capabilities. However, since the effects of plyometric training were not covered by the current study, these conclusions remain speculative. Accordingly, further research on the effect of plyometric training in rowers with a deficit in the field of reactive force capabilities and the application of slow SSC exercises is needed.

The main limitation of the present study is that no SSC of the fascicle has yet been detected or investigated in rowing. However, the following four aspects suggest SSC mechanisms in rowing: (a) The sequence of flexion and extension (of the legs) during one rowing cycle, (b) the kinematic observations that this FEC movement can be performed in rowing as fast as in countermovement jumps (Held et al., 2019, 2020), (c) the muscle activity during the late slide phase prior to the onset of a new rowing stroke (Janshen et al., 2009; Guével et al., 2011; Turpin et al., 2011; Fleming et al., 2014; Shaharudin et al., 2014; Held et al., 2020), and (d) the confirmation of the SSC typical performance enhancement during FEC-type rowing. Altogether, future research should precisely determine whether the muscle fasciae complete an SSC during rowing and investigate the verification of the SSC in rowing. In this context, sEMG, goniometer, and ultrasound measurements of the fascicle’s operating length and velocity as well as the activation of a leg extensor muscle during rowing are currently in preparation. Additionally, further research is needed on the extent to which the storage (and delivery) of elastic energy (induced by muscle preactivation during the eccentric phase) (Kubo et al., 1999; Bojsen-Møller et al., 2005) and the stretch-induced increase in contractility during the concentric phase (Rode et al., 2009; Seiberl et al., 2015) contribute to performance enhancement during FEC-type rowing. One methodological limitation of the current study is that the accuracy of the motion-capturing method depends on the examiner (Suleder, 2010). To increase the accuracy, markers were additionally used in order to identify the handle and seat position, and the handle motion takes place on the same level (distance to the camera) as the seat motion. In addition, the accuracy of the motion-capturing system was compared with the handle displacement data of the FES setup (error of measurement <2%). Accordingly, the accuracy of the motion-capture method can be considered as appropriate in the current study. The rowing and P_leg_ determination in the current paper was based on the handle force and the handle pathway (Fukunaga et al., 1986; Dal-Monte and Komo, 1989; Zatsiorsky and Yakunin, 1991). Since the rower applies power at the handle and the foot stretcher, stretcher force is useful for the P_row_ determination (Kleshnev, 2016). Nevertheless, conclusions can also be drawn without the stretcher force, as the calculated P_row_ is the only propulsive energy source of the rower-boat system (Kleshnev, 2016). In this context, the proportion of P_leg_ on the total P_row_ was further determined based on the handle force and the leg (seat) movement speed (Kleshnev, 2016; Held et al., 2019). However, there is some movement of the hips relative to the seat, resulting in small leg speed deviations. Overall, this error can be classified as minimal because the extent of the hip movement (relative to the seat) is negligible (<2% of the total seat movement amplitude) (Held et al., 2019).

In conclusion, the current research clearly showed that an FEC led to notably higher handle force, WPS, P_row_, and P_leg_ outputs compared to isolated concentric rowing movement. These findings are in line with the general force, work, and power enhancement in an SSC (Cavagna et al., 1968; Bosco et al., 1987; Gregor et al., 1988). Taking the observed sEMG activity during the late slide phase prior to the onset of a new rowing stroke into account (Janshen et al., 2009; Guével et al., 2011; Turpin et al., 2011; Fleming et al., 2014; Shaharudin et al., 2014; Held et al., 2020), the current results deliver meaningful insights into force enhancement enabling an adequate FEC during rowing patterns. Future ultrasound studies should investigate the occurrence and magnitude of potential SSC in rowing.

## Data Availability Statement

All datasets generated for this study are included in the article/Supplementary Material.

## Ethics Statement

The studies involving human participants were reviewed and approved by the Ethikkommission – Deutsche Sport Hochschule Köln. The patients/participants provided their written informed consent to participate in this study.

## Author Contributions

SH, TS, and LD have planned the study and together developed the final manuscript. SH conducted the study.

## Conflict of Interest

The authors declare that the research was conducted in the absence of any commercial or financial relationships that could be construed as a potential conflict of interest.
