# Supplementary material for: 10% Higher Rowing Power Outputs After Flexion-Extension-Cycle Compared to an Isolated Concentric Contraction in Sub-Elite Rowers
Source: Front Physiol. 2020 Jun 17;11:521. doi: 10.3389/fphys.2020.00521 (PMC7311752; doi:10.3389/fphys.2020.00521)
Supplement: Supplementary file 1 [file Table_1.DOCX]

**Supplemental Table 1.** Overview of the concentric (DRIVE), isometric pre-contraction (ISO-DRIVE), and FEC type (SLIDE-DRIVE) rowing-trials data (mean ± standard deviation). P_row_: mean mechanical rowing-power, P_leg_: leg-power, WPS: work-per-stroke, F_max_: maximal handle force during drive, T_drive_: duration of drive, L_drive_: length of drive, L_slide_: length of seat-motion, v_drive_: average handle speed during drive. The significances (***: p < .001, **: p < .01, *: p < .05) and effect sizes (†††: η_p_^2^ ≥ .138, ††: η_p_^2^ ≥ .059, †: η_p_^2^ ≥ .01) of the repeated measurement ANOVA are given. In addition, significances of Bonferroni post-hoc tests (p) and effect-size (+++: SMD > |.8|, ++: SMD > |.5|, +: SMD > |.2|) of DRIVE- to ISO-DRIVE-measurements (DRIVE – ISO-DRIVE), DRIVE- to SLIDE-DRIVE-measurements (DRIVE – SLIDE-DRIVE) and ISO-DRIVE- to SLIDE-DRIVE-measurements (ISO-DRIVE – SLIDE-DRIVE) are presented.

|  | | **P_row_ (W)** | **P_leg_ (W)** | **WPS (J)** | **F_max_ (N)** | **T_drive_ (s)** | **L_drive_ (m)** | **L_slide_ (m)** | **v_drive_ (m/s)** |
| --- | --- | --- | --- | --- | --- | --- | --- | --- | --- |
| **DRIVE** | | 441  ± 156 | 145  ± 58 | 1157  ± 195 | 1154  ± 192 | 1.24  ± 0.12 | 1.373  ± 0.048 | 0.519  ± 0.049 | 1.12  ± 0.09 |
| **ISO-DRIVE** | | 465  ± 142 | 157  ± 52 | 1203  ± 230 | 1149  ± 190 | 1.23  ± 0.08 | 1.416  ± 0.067 | 0.549  ± 0.081 | 1.15  ± 0.08 |
| **SLIDE-DRIVE** | | 485  ± 147 | 171  ± 63 | 1277  ± 251 | 1205  ± 200 | 1.22  ± 0.09 | 1.459  ± 0.069 | 0.571  ± 0.124 | 1.20  ± 0.09 |
| **ANOVA** | **p** | *** | *** | *** | *** |  | *** | ** | *** |
|  | ***η*_p_^2^** | ††† | ††† | ††† | ††† | † | ††† | †† | ††† |
| ***Bonferroni* post-hoc tests (p)** | **DRIVE – ISO-DRIVE** |  |  |  |  |  |  |  |  |
|  | **DRIVE – SLIDE-DRIVE** | *** | *** | *** | *** |  | *** |  | *** |
|  | **ISO-DRIVE – SLIDE-DRIVE** |  |  | * | *** |  | * |  | *** |
| **SMD** | **DRIVE – ISO-DRIVE** |  | + | + |  |  | +++ | + | + |
|  | **DRIVE – SLIDE-DRIVE** |  | + | + |  |  | +++ | + | + |
|  | **ISO-DRIVE – SLIDE-DRIVE** |  | + | + | + |  | ++ | + | ++ |
| **Percentage increase** | **DRIVE – ISO-DRIVE** | 6,7   ± 13,4 % | 11,4  ± 22,9 % | 3,8  ± 8,3 % | -0,3  ± 5,9 % | -0,2  ± 5,7 % | 3,2  ± 4,8 % | 5,9  ± 13,4 % | 3,4  ± 4,9 % |
|  | **DRIVE – SLIDE-DRIVE** | 11,8  ± 14,0 % | 19,6  ± 26,7 % | 9,9  ± 10,5 % | 4,4  ± 7,0 % | -1,0  ± 5,5 % | 6,3  ± 4,8 % | 10,9  ± 23,2 % | 7,6  ± 6,0 % |
|  | **ISO-DRIVE – SLIDE-DRIVE** | 6,0   ± 16,0 % | 9,8  ± 26,6 % | 6,1  ± 6,7 % | 5,0  ± 4,8 % | -0,9  ± 4,5 % | 3,2  ± 5,2 % | 4,9  ± 2,8 % | 4,2  ± 4,0 % |
